# Improvement of the Resuscitation Environment with the Modified Toyota Kaizen Approach Via *In Situ* Anaesthesia Simulation Training

**DOI:** 10.4274/TJAR.2024.241598

**Published:** 2024-09-17

**Authors:** Taiki Kojima, Shogo Ichiyanagi, Mitsunori Miyazu

**Affiliations:** 1 Aichi Children’s Health and Medical Centre, Department of Anaesthesiology, Aichi, Japan

*In situ* anesthesia simulation is performed by a multidisciplinary team (anaesthesiologists and nurses) with good team dynamics and clinical and resource management skills.^1^ However, in current *in situ* simulations, opportunities to discuss the environmental aspects of resuscitation during anaesthetic emergencies is limited. Optimizing the resuscitation environment (optimal allocation or amount of prepared resuscitation equipment in operating rooms and routes for effective resuscitation) can reduce the stress levels of resuscitation team members and ensure smooth resuscitation during anaesthetic emergencies. However, current resuscitation guidelines lack detailed guidance on how to effectively optimize equipment preparation.

Kaizen, meaning “continuous improvement” or “change for the better,” is based on the idea that small incremental changes and improvements can lead to significant advancements over time. Toyota Motor Corporation (Tokyo) incorporates Kaizen principles into its production and operations processes. Kaizen has recently been adopted in various healthcare fields worldwide for process improvement, error reduction, patient safety, and staff training and education.^2, 3^ It reduces stress and increases worker satisfaction by creating a pleasant working environment.^4^ During resuscitation in operating suites, stressful situations, such as the unavailability of necessary medications and equipment or blocked access to patients, are encountered due to a lack of streamlined routes for team members. Solving these issues can improve the quality of resuscitation during chaotic anaesthesia emergencies in operating suites.

The “3As” key components of Kaizen were selected from five standardizations (“Access,” “Amount,” “Allocation,” “Naming,” and “Coloring”) based on the original Toyota Kaizen method for resuscitation in operating suites. First, “Access” focuses on optimal routes for smoothly channeling necessary medications and equipment. Second, “Amount” ensures appropriate numbers of medications and equipment for efficient resuscitation. Finally, “Allocation” standardizes the location of medications and equipment in each operating suite.

Our institution incorporated the Kaizen method into *in situ* simulation debriefing to improve the environment for efficient resuscitation and reduce the stress levels of the resuscitation team members. Simulation educators can set up an *in situ* simulation that intentionally modifies the “3 As” of medical equipment and medications in operating rooms. These situational changes can provide learners with critical learning experiences regarding the importance of the “3 As” and offer them opportunities to explore potential improvements in current emergency equipment preparation within their institutions. A Kaizen consultant identified issues and provided feedback to change the resuscitation environment and improve resuscitation quality.

Changes to the resuscitation environment included: 1) access to team members during resuscitation, 2) optimal amount of resuscitation equipment in the emergency cart and operating suite, and 3) allocation of resuscitation equipment (airway securing equipment, medications). Moreover, the facilitator encouraged the resuscitation team members to discuss potential environmental issues during the debrief. Additionally, the facilitators should educate learners on the Kaizen method and clearly explain the role of the Kaizen consultant during the debriefing session.

In conclusion, there is a lack of focus on resuscitation-related environmental aspects in simulation training, despite their potential influence on the quality of resuscitation and team stress levels. This limitation can be addressed using the Kaizen approach.
